# Surveillance and genetic data support the introduction and establishment of *Aedes albopictus* in Iowa, USA

**DOI:** 10.1038/s41598-022-06294-5

**Published:** 2022-02-08

**Authors:** David R. Hall, Ryan E. Tokarz, Eleanor N. Field, Ryan C. Smith

**Affiliations:** 1 Department of Entomology, Iowa State University, Ames, IA USA; 2 Present Address: Department of International and Global Studies, Mercer University, Macon, GA USA

**Keywords:** Ecosystem ecology, Invasive species, Population dynamics

## Abstract

*Aedes albopictus* is a competent vector of several arboviruses that has spread throughout the United States over the last three decades. With the emergence of Zika virus in the Americas in 2015–2016 and an increased need to understand the current distributions of *Ae. albopictus* in the US, we initiated surveillance efforts to determine the abundance of invasive *Aedes* species in Iowa. Here, we describe surveillance efforts from 2016 to 2020 in which we detect stable and persistent populations of *Aedes albopictus* in three Iowa counties. Based on temporal patterns in abundance and genetic analysis of mitochondrial DNA haplotypes between years, our data support that *Ae. albopictus* are overwintering and have likely become established in the state. The localization of *Ae. albopictus* predominantly in areas of urbanization, and noticeable absence in rural areas, suggests that these ecological factors may contribute to overwintering success. Together, these data document the establishment of *Ae. albopictus* in Iowa and their expansion into the Upper Midwest, where freezing winter temperatures were previously believed to limit their spread. With impending climate change, our study provides evidence for the further expansion of *Ae. albopictus* into temperate regions of the United States resulting in increased risks for vector-borne disease transmission.

## Introduction

*Aedes albopictus* is an invasive mosquito species that in recent decades has spread across multiple continents predominantly through global trade^1–4^. With the first report of its establishment in the United States (US) in Texas in 1985^5^, its range has continually expanded to more than 26 states, gradually spreading northward and westward across the US^6–8^ with likely further expansion fueled by climate change and increasing urbanization^3^. With anthropophilic feeding behavior^4,9^ and as a competent vector of at least 26 mosquito-borne arboviruses^10^, the introduction of *Ae. albopictus* into new locations in the US raises a significant public health concern.

With *Ae. albopictus* as a competent vector of Zika virus (ZIKV)^11,12^, the emergence of ZIKV in the Americas in 2015 and 2016 created a critical need to better understand the distributions of *Ae. albopictus* in the US in order to determine the potential risks for ZIKV transmission. Previous studies have detected *Ae. albopictus* populations across the Midwest^7,8,13–17^. This includes Iowa, which has seen sporadic detections of *Ae. albopictus* that likely represent rare and unsuccessful introduction events^17^. However, with established populations of *Ae. albopictus* in the neighboring states of Missouri^7,8,13^ and Illinois^7,8,16^, there is a high likelihood of additional *Ae. albopictus* introduction events and potential invasion as models suggest that Iowa is within this species’ predicted range^18^. Initial targeted surveillance in 2016 along the southern Iowa border failed to detect *Ae. albopictus*^19^, yet with sampling only over a single year, there may not have been adequate trapping efforts to identify low-density populations.

In this study, we describe our continued monitoring of mosquito populations in Iowa through targeted surveillance efforts focusing on invasive *Aedes* species. Expanding on our initial efforts^19^, we used a trapping network consisting of BG sentinel and Gravid *Aedes* traps from 2017 to 2020 to monitor mosquito populations in a total of 25 counties over a 5-year period (2016–2020). Through these efforts, we document the detection and likely establishment of *Ae. albopictus* in three Iowa counties. In addition, we provide evidence of their intra-county movement and the ecological variables that define their presence and absence, elucidating the potential ecological barriers that have thus far prevented their further spread to adjacent counties. Genetic analysis confirms the subsistence of genetic haplotypes between years, supporting the establishment of *Ae. albopictus* in each of the respective counties in which it has been detected, providing insight into the origins of their introduction.

## Results

### Mosquito surveillance and detection of *Ae. albopictus* in Iowa

**Figure 1:**
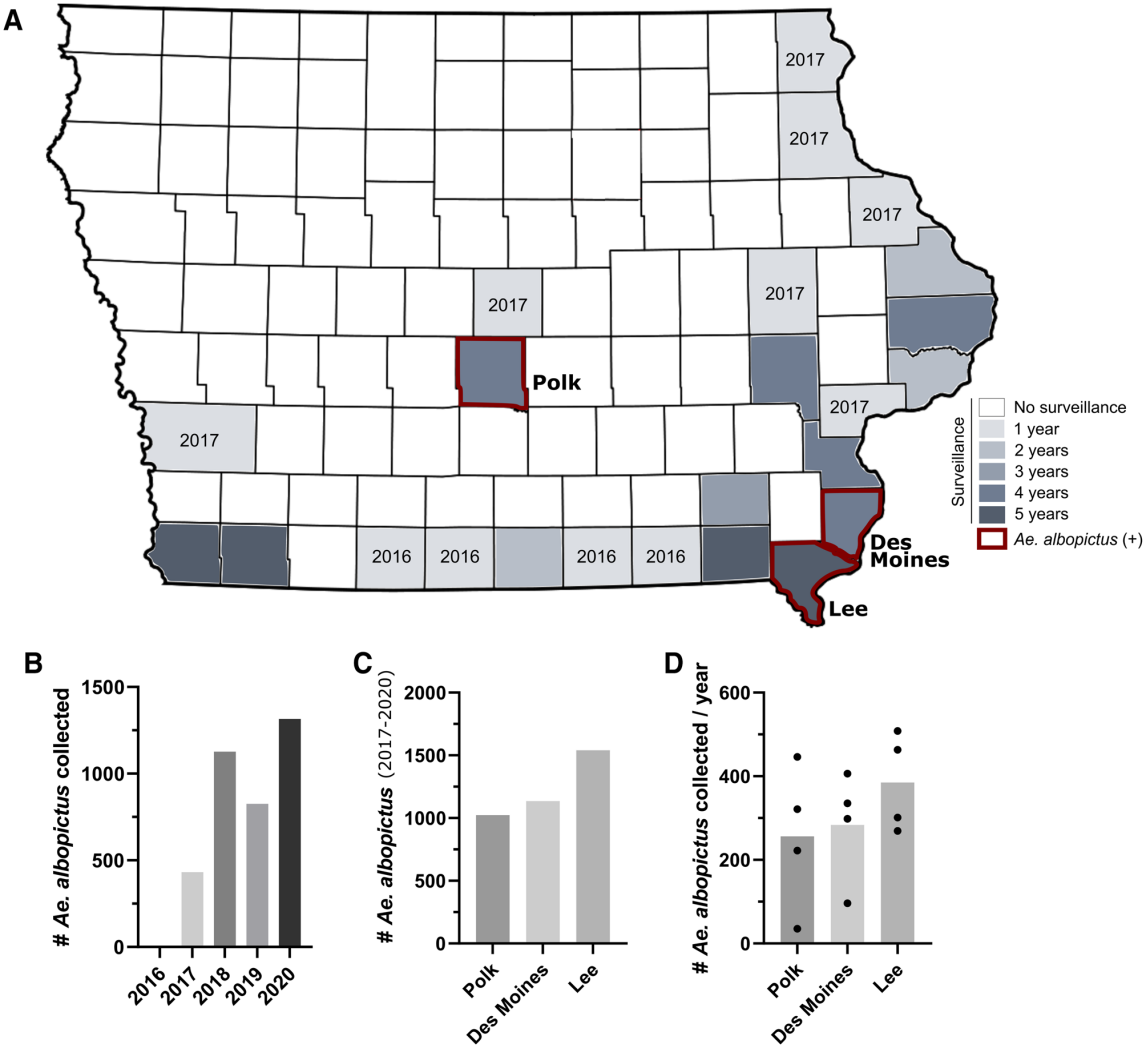
Overview of mosquito trapping locations and number of *Ae. albopictus* collected in Iowa (2016–2020). Mosquito surveillance efforts targeting invasive *Aedes* species were performed in a total of 25 Iowa counties from 2016 to 2020 (**A**). Participating counties were shaded based on the number of years trapping efforts were performed in that county, with years listed when a county was included in trapping efforts for only a single year. Counties in which *Ae. albopictus* were detected are outlined in red. The number of *Ae. albopictus* collected are displayed for each year (**B**), in each *Ae. albopictus*-positive county (**C**), and average number collected per year in each county (**D**).

To determine if the invasive mosquito species, *Ae. aegypti* and *Ae. albopictus*, could be found in the state of Iowa, we performed targeted mosquito surveillance in a total of 25 counties from 2016 to 2020 (Fig. 1A, Table S1, Table S2). After initial surveillance efforts along the southern border of the state in 2016^19^, we extended our trapping efforts from 2017 to 2020 to more densely populated counties and to those bordering the Mississippi River (Fig. 1A, Table S1, Table S2). Although *Ae. aegypti* and *Ae. albopictus* were not detected in 2016^19^, a total of 432 *Ae. albopictus* were collected in 2017 from three Iowa counties (Polk, Lee, and Des Moines) (Fig. 1A, B). In subsequent years (2018–2020), *Ae. albopictus* were similarly detected in each of the same three counties in increased numbers, reaching a high of 1315 *Ae. albopictus* detected in 2020 (Fig. 1B). From 2017 to 2020, a total of 3700 adult *Ae. albopictus* were collected, with Lee County displaying the highest total of *Ae. albopictus* amongst the three counties (Fig. 1C) and consistently producing the highest number of *Ae. albopictus* between years (Fig. 1D). Together, these data suggest that in recent years *Ae. albopictus* have been introduced into the state, and have potentially become established in three Iowa counties.

### *Ae. albopictus* population dynamics support their ability to overwinter in Iowa

**Figure 2:**
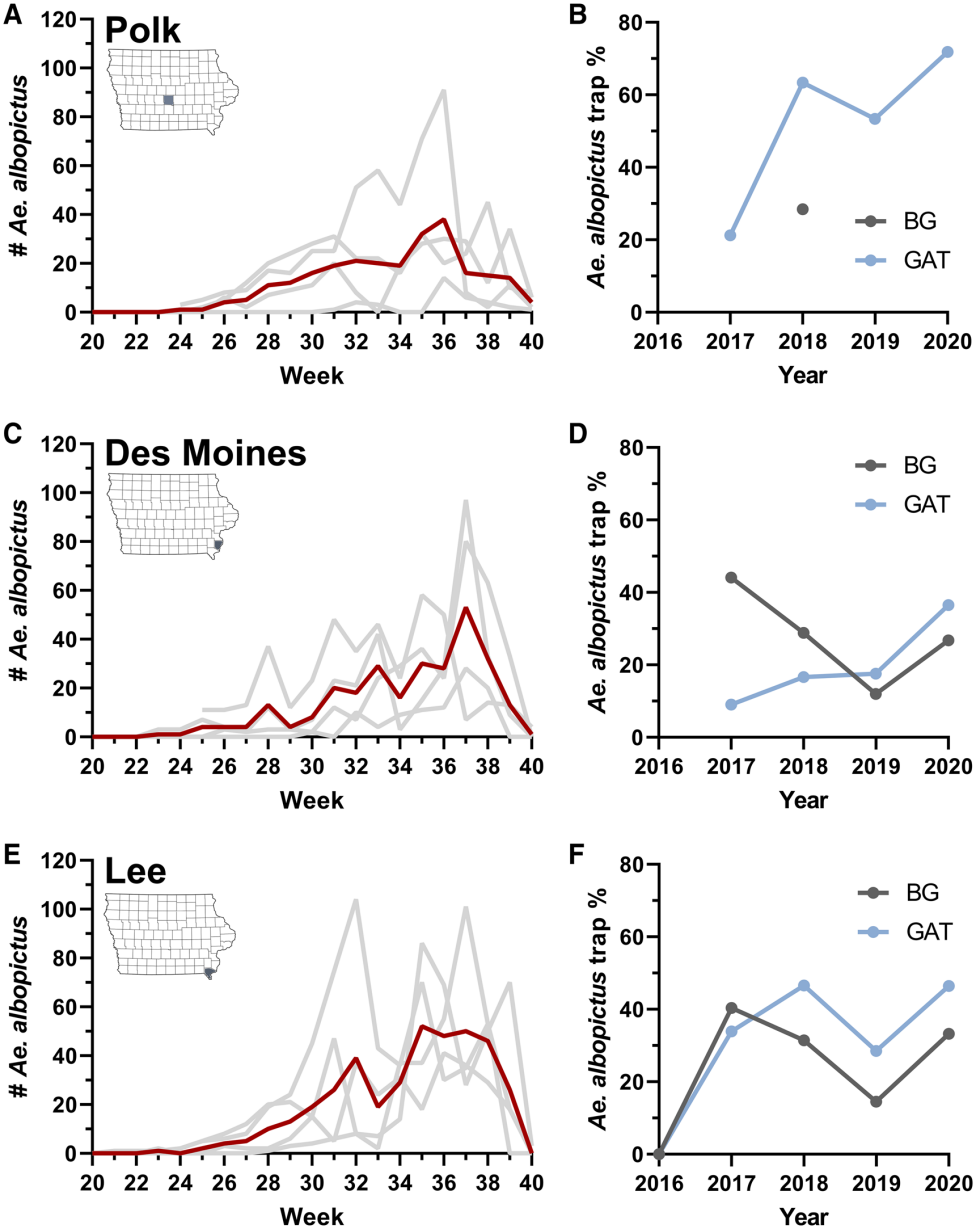
Abundance of *Ae. albopictus* in each positive Iowa county. The temporal abundance and percentage of *Ae. albopictus* in overall trap yields is displayed for Polk (**A, B**), Des Moines (**C, D**) and Lee County (**E, F**). Temporal abundance for each county (**A, C, E**) is displayed by epidemiological week, with the average abundance (2017–2020) displayed in red, while individual years are denoted by light gray lines. The percentage of *Ae. albopictus* collected of the total trap yields (**B, D, F**) are displayed for BG Sentinel (BG) and Gravid *Aedes* traps (GAT) for each year.

To provide further support that *Ae. albopictus* have become established in each of the three counties, we examined *Ae. albopictus* weekly numbers and overall contributions to trapping yields within each of the mosquito trapping seasons from 2016 to 2020 in counties for which *Ae. albopictus* were detected (Fig. 2). Across counties, *Ae. albopictus* populations reached peak abundance in late summer (late August, early September; approximately epidemiological weeks 35 and 36), followed by sharp declines in abundance by early October (week 40) (Fig. 2). For Polk County in central Iowa, *Ae. albopictus* was first detected in week 31 (early August) in 2017, yet in subsequent years (2018–2020) were consistently identified in mid-June (weeks 24 and 25; Fig. 2A). Moreover, *Ae. albopictus* represented a much larger percentage of overall trap yields between 2018–2020 when compared to 2017 (Fig. 2B), suggesting that their earlier abundance and higher proportion in the collected samples are indicative of their potential establishment.

Similar patterns of *Ae. albopictus* abundance were also recorded in the southeastern portion of the state in Des Moines and Lee counties (Fig. 2). For Des Moines County, the first detection of *Ae. albopictus* in 2017 occurred in week 30 (late July), while they were regularly detected in June (weeks 23–26) in subsequent years (2017–2020; Fig. 2C). Although sites in Des Moines County displayed some variability between trap types, *Ae. albopictus* comprised between ~ 10–40% of the overall number of mosquitoes collected in the county (Fig. 2D). Lee County recorded the earliest detection of *Ae. albopictus* in 2017, with the first samples identified in week 28 (mid-July). In the years following (2018–2020), *Ae. albopictus* were found as early as week 21 (late-May; Fig. 2E). Aside from 2016 when *Ae. albopictus* were not detected in our initial trapping efforts^19^, *Ae. albopictus* represented ~ 34% of the total trap yields when averaged across years and trap types (Fig. 2F). While we account for some yearly variation in occurrence and overall abundance, these data provide further support for the overwintering and establishment of *Ae. albopictus* in multiple Iowa counties.

### Influence of landscape ecology on *Ae. albopictus* abundance

To determine if landscape ecology influences the presence of *Ae. albopictus* in Iowa, we looked to more closely examine the trapping site locations in each of the *Ae. albopictus-*positive counties. Trapping efforts in Polk County consisted of only a single site in close proximity to a facility involved in tire transport, with surrounding areas serving as an ideal habitat for *Ae. albopictus* (abundant larval habitats, tree cover, access to diverse hosts; Figure S1). Additional focused trapping efforts (BGs, GATs) were not performed at other locations in Polk County during this study. However, non-targeted surveillance involved with our West Nile virus surveillance program using other trap types (New Jersey Light Traps and Frommer Updraft Gravid Traps) have detected low numbers of *Ae. albopictus* in 2019 and 2020 at nearby locations in Polk County (Figure S2). The detection of *Ae. albopictus* in these suburban environments at locations that have been continuously surveyed since 2016, suggests that *Ae. albopictus* have likely dispersed greater than 3 miles from their presumed point of introduction in recent years, providing further support for the ability of *Ae. albopictus* to overwinter in Polk County.

**Figure 3:**
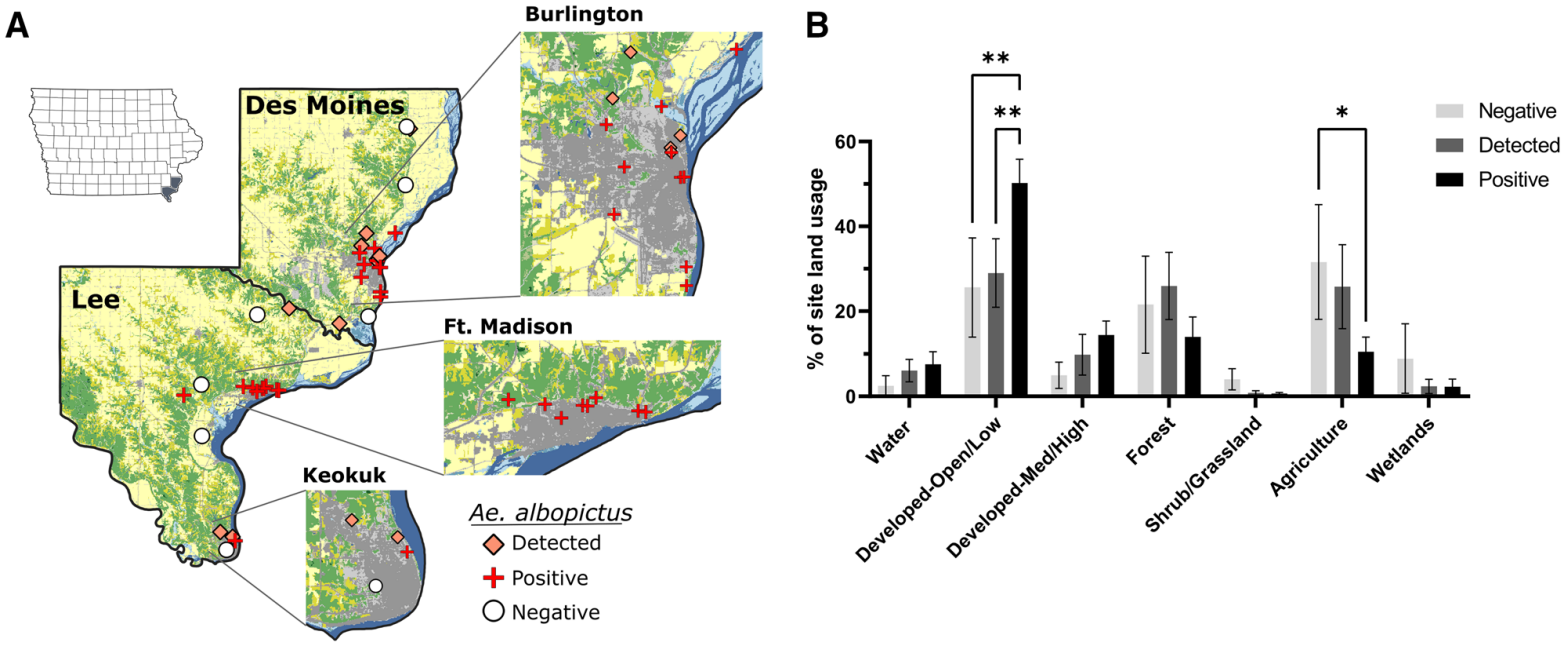
Landscape ecology influences *Ae. albopictus* abundance. Trapping site locations for both Des Moines and Lee counties display the presence/absence of *Ae. albopictus* as either positive (detected every year; red cross), detected (detected in some years; orange diamond), or negative (never detected; white circle) (**A**). Insets display expanded views for the most populous cities in Des Moines (Burlington) and Lee County (Ft. Madison and Keokuk). To better understand differences in the ecology of sites where *Ae. albopictus* were positive, detected, or negative, land use/land cover analysis was performed using 500 m radius around each trapping location and displayed for different land use/land cover classifications (**B**). Statistical analysis was performed using a 2-way ANOVA and a Tukey’s multiple comparison test using GraphPad Prism software. Asterisks denote significance (*p < 0.05; **p < 0.01). The map was created in QGIS version 3.14.1 (www.qgis.org) using an Iowa county boundary shapefile obtained from Iowa Geospatial Data (https://geodata.iowa.gov) and land cover data obtained from the Multi-Resolution Land Characteristics Consortium (MRLC) National Land Cover Database (NLCD) (https://www.mrlc.gov/). The image was produced using the layout manager tool within QGIS and edited using Inkscape (https://inkscape.org/).

In contrast to the likely introduction of *Ae. albopictus* in Polk County associated with tire transport, there were no obvious mechanisms for the introduction of *Ae. albopictus* in Des Moines and Lee counties. As a result, the multiple trapping locations in Des Moines and Lee counties provided a better opportunity to determine the influence of landscape ecology on the presence or absence of *Ae. albopictus* (Fig. 3). To address this question, we performed comparative land cover analysis on a total of 37 trapping site locations for which we defined each site for the presence/absence of *Ae. albopictus* (Fig. 3, Table S3). A total of 22 sites where *Ae. albopictus* were detected every year were considered “positive”, while the six sites for which *Ae. albopictus* were never found were considered “negative” (Table S3). Nine other sites where *Ae. albopictus* were identified but not collected every year were defined as “detected” (Table S3), suggesting that these sites represent new introductions that may or may not support established populations. Each of these trapping sites were mapped to their respective locations in Des Moines and Lee counties (Fig. 3A), and the landscape ecology of “positive”, “negative”, and “detected” sites were compared (Fig. 3B). We identified that areas of low-density development were significantly correlated with the presence of *Ae. albopictus*, while the percentage of agricultural areas were negatively associated with the presence of *Ae. albopictus* (Fig. 3B). These data are supported by the spatial locations of the trapping site locations, where sites within urbanized areas were predominantly positive, while those located in more rural areas were typically negative (Fig. 3A). This corresponds with the preferred habitat of *Ae. albopictus* which is most commonly associated with urban and suburban environments^20,21^.

Furthermore, our trapping data provide support for the expansion of *Ae. albopictus* in both Des Moines and Lee counties. The presence of several sites for which *Ae. albopictus*, were detected, but not necessarily established, supports the movement of this mosquito species into new areas. This is further evidenced by the presence of *Ae. albopictus* in Keokuk (Lee County; Fig. 3A) in 2018, where previous trapping efforts in 2016^19^ and 2017 suggested that these mosquito species were noticeably absent. This contrasts surveillance in Ft. Madison (Lee County; Fig. 3A) where *Ae. albopictus* have been detected at every site since 2017 (Fig. 3A, Table S3). With these two cities separated by ~ 16 miles, these data imply that the distribution of *Ae. albopictus* in Lee County may be continually expanding into new locations.

### Genetic haplotype analysis identifies likely sources of introduction and supports overwintering of *Ae. albopictus* in Iowa

**Figure 4:**
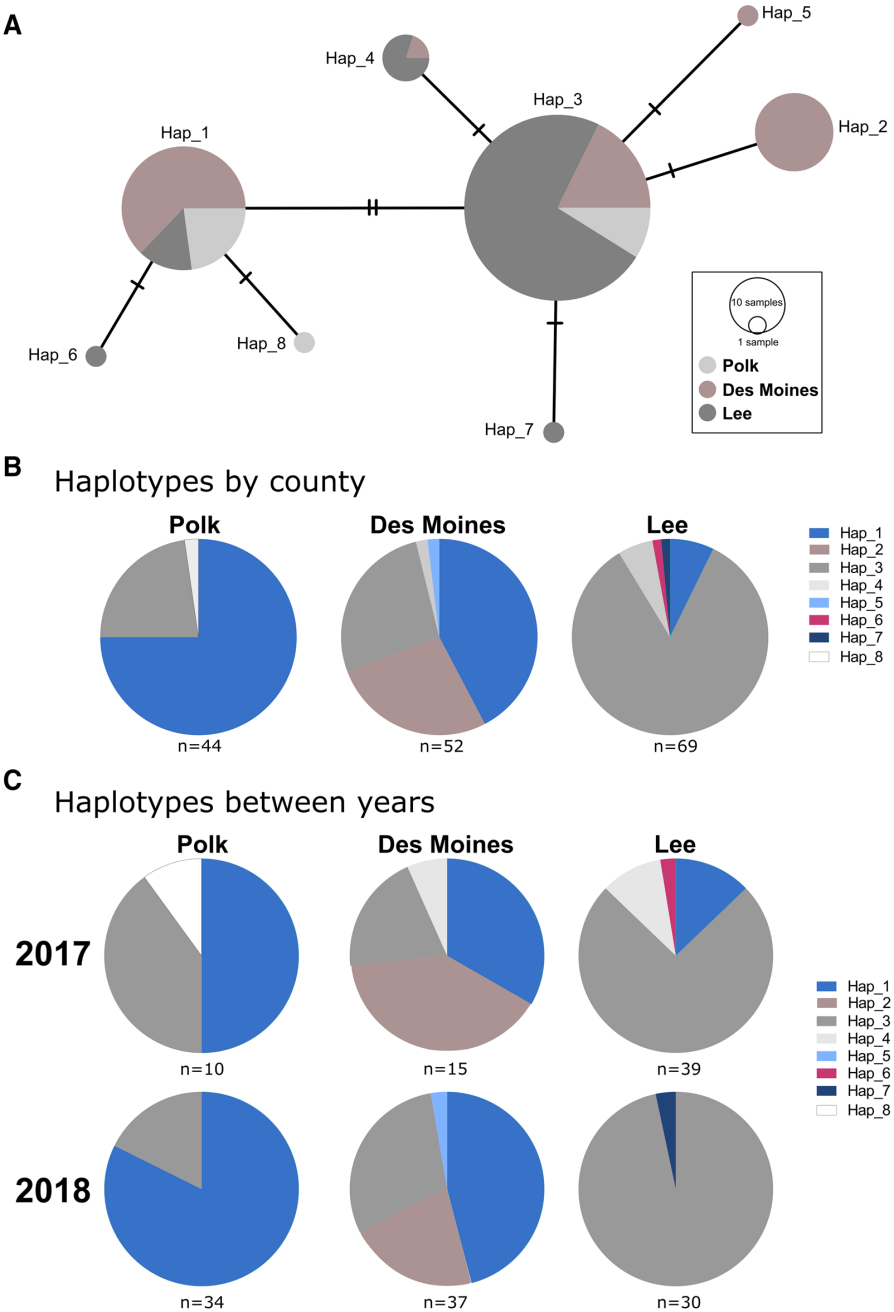
Comparative analysis of *Ae. albopictus* mtDNA haplotypes identified in Iowa. Comparisons of *Ae. albopictus* mtDNA haplotypes identified in Iowa are displayed as circles, with dashes on connecting lines indicating the number of nucleotide differences between haplotypes (**A**). Circle size corresponds to the number of individual samples, with differences in color representing the proportion samples from each respective county. Additional pie charts display differences in haplotypes between each *Ae. albopictus*-positive county (**B**) and the persistence of haplotypes between years (**C**).

To better understand the origins of the *Ae. albopictus* collected in each of the three positive counties, DNA was isolated from a total of 165 individual samples and sequences of the mitochondrial CO1 gene were analyzed similarly to previous studies^16,22^. Sequence analysis resulted in the identification of 8 genetic haplotypes (Fig. 4A, Table S4), distinguished by single nucleotide polymorphisms ranging between one to three nucleotides (Fig. 4A, Table S5). The most abundant haplotypes, *hap_1* and *hap_3*, are distinguished by two nucleotides and were found in each of the three *Ae. albopictus* positive counties (Fig. 4A). Both haplotypes represent common DNA haplotypes detected in other locations in the United States^16,22^, southeast Asia^22,23^, and Europe^22^ (Table S4). Moreover, *hap_2* and *hap_3* were previously detected in Illinois^16^ (Table S4), which due to its close proximity suggests that Illinois may serve as the likely origin and source for the introduction of *Ae. albopictus* in Iowa.

For the three counties where *Ae. albopictus* was detected, three or more haplotypes were detected, with both Des Moines and Lee counties displaying a total of five haplotypes (Fig. 4B). While the majority of samples in Polk and Lee counties represented a single haplotype, with *hap_1* and *hap_3* respectively the most abundant in Polk and Lee counties (Fig. 4B). Des Moines County displayed the most diverse population with *hap_1*, *2*, and *3* comprising the majority of samples (Fig. 4B). When samples were examined between years (2017 and 2018), the predominant haplotype(s) were consistent between years in each county where *Ae. albopictus* were detected (Fig. 4C), providing further support for the establishment of these populations in each of the respective counties.

### *Ae. albopictus* overwintering in below freezing winter isotherms

**Figure 5:**
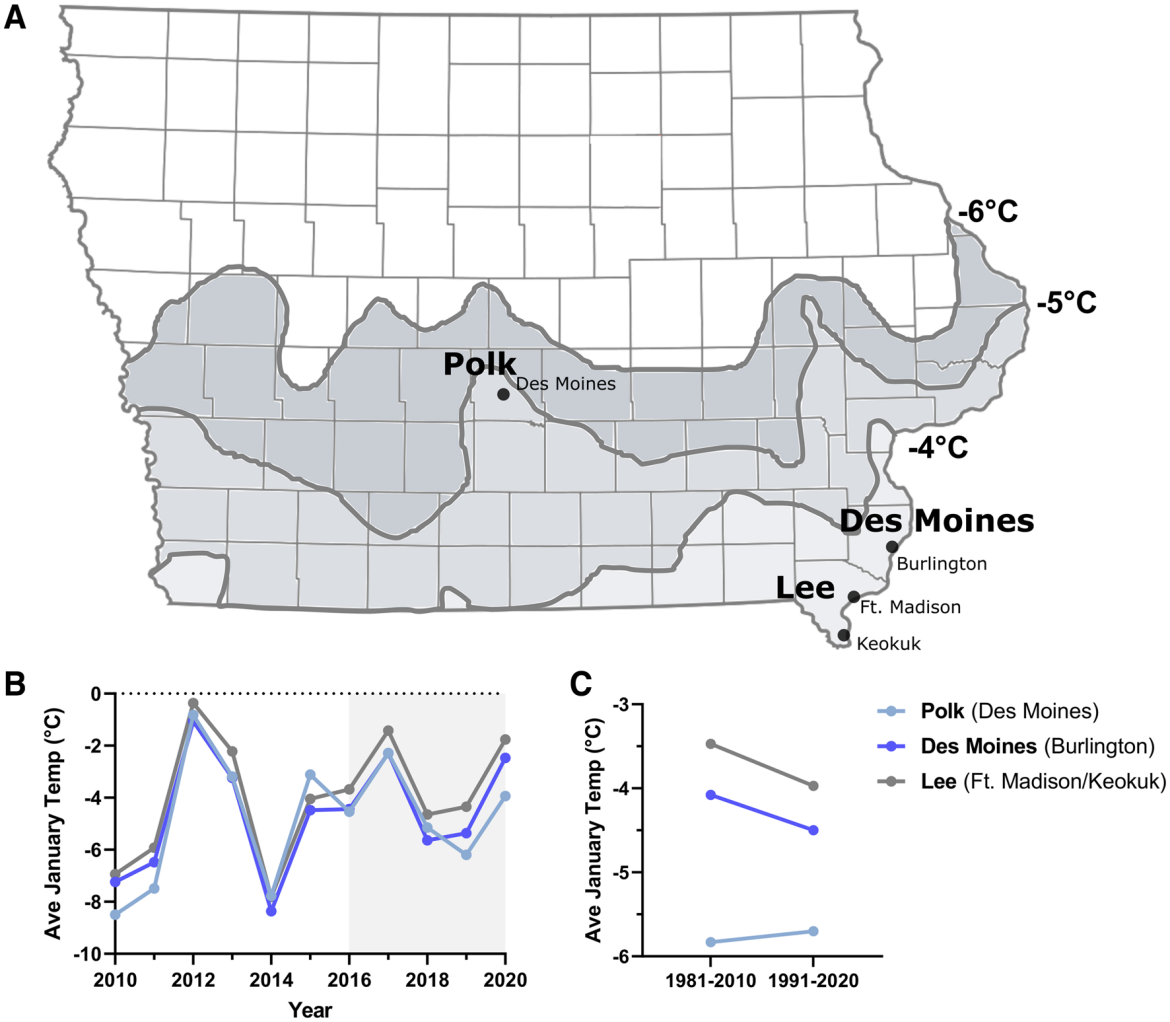
Overwintering temperatures in Iowa. Average winter (December, January, February) temperatures are displayed for Iowa (**A**). Shaded regions represent different temperature isotherms. Cities and counties where stable populations of *Ae. albopictus* have been detected are shown. Annual January temperatures are displayed from 2010 to 2020 to indicate differences in yearly temperatures for each respective *Ae. albopictus*-positive county (**B**). The shaded region from 2016 to 2020 represent the study period where targeted trapping efforts have focused on invasive *Aedes* species. Differences in January temperatures from the 30-year average from 1981–2010 and 1991–2020 examine the potential impacts of climate change on overwintering temperatures (**C**).

Based on our surveillance data (Fig. 2) and genetic analysis of DNA haplotypes (Fig. 4), our results provide a strong argument for the introduction and establishment of *Ae. albopictus* in Iowa. Since overwintering temperatures have largely been attributed to limiting the spread of *Ae. albopictus* in North America^24^, we examined the average winter temperature (December, January, February) isotherms for Iowa (1981–2010). Each of the *Ae. albopictus*-positive counties have average winter temperatures below freezing, with Des Moines and Lee counties in the − 3 to − 4 °C isotherm, and Polk County in the − 4 to − 5 °C isotherm (Fig. 5A). While January temperatures (typically the coldest month) vary between years, temperatures ranged between − 1 and − 6 °C in the *Ae. albopictus*-positive counties during our study period (Fig. 5B). These low temperatures are traditionally considered to be not conducive to *Ae. albopictus* overwintering^18,24^, suggesting that the *Ae. albopictus* populations in Iowa have been able to adapt to these freezing temperatures or have found adequate insulated environments to survive the winter. To determine whether global warming may have elevated recent winter temperatures and increased the likelihood that *Ae. albopictus* could successfully overwinter in Iowa, we examined the 30-year average January temperatures from 1981–2010 and 1991–2020 (Fig. 5C). While Polk County displayed slightly warmer temperatures in recent years, temperatures in both Des Moines and Lee counties were ~ 0.5 °C cooler (Fig. 5C), arguing that the recent expansion of *Ae. albopictus* into these areas is not the result of higher winter temperatures.

## Discussion

While limited detections of *Ae. albopictus* in Iowa (12 total from 1999 to 2016) have previously been described^17,25^, these rare incidents were likely the result of isolated introduction events. However, with the introduction of Zika virus in the Americas in 2015–2016, there was an increased need to define the range of competent *Aedes* vectors throughout the US. Although our initial efforts in 2016 along the southern Iowa border did not detect *Ae. albopictus*^19^, the results of our expanded surveillance efforts from 2017 to 2020 presented here describe the detection of more than 3700 *Ae. albopictus* adult samples from three Iowa counties.

From these data, several lines of evidence support the establishment of *Ae. albopictus* in Iowa. This includes the consistent, early-season detection of *Ae. albopictus* in May and June, as well as the high percentage of the overall trapping yields for each of the three *Ae. albopictus*-positive counties. This is further validated by the occurrence of consistent mtDNA haplotypes between years, indicative of stable, genetic populations of *Ae. albopictus* that support mosquito overwintering.

While we cannot fully eliminate the possibility that new, annual introductions may also contribute to the *Ae. albopictus* samples that were collected, the high number of individual adult mosquito samples collected from each *Ae. albopictus*-positive county makes this possibility unlikely. Although the primary site examined in Polk County is associated with tire transport, the surrounding areas are ideal *Ae. albopictus* habitat, with adequate tree cover, the presence of human and mammalian hosts, and an abundance of human-derived containers/tires that can serve as sites for oviposition that would support *Ae. albopictus* in much greater density. Although no obvious mechanisms of introduction by tire transport have been determined for Des Moines and Lee counties, the proximity to the Mississippi River and interstate highways that support human-associated transport likely account for the large number of *Ae. albopictus* collected in both locations. However, the consistency between years and the representation of similar *Ae. albopictus* mtDNA haplotypes across each county suggest that these are stable populations, supporting that the overwintering and establishment of *Ae. albopictus* as the most likely cause for the mosquitoes collected during our study period.

The presumed establishment of *Ae. albopictus* in Iowa challenges previous studies that have suggested that *Ae. albopictus* populations rarely extend north of 40°N latitude^7,18^, presumably due to sub-zero winter temperatures that limit the ability of *Ae. albopictus* to overwinter in North America^24,26^. With average winter temperatures below 0 °C which have traditionally limited overwintering and the expansion of *Ae. albopictus*^26^, the freezing winter conditions in Iowa have traditionally been viewed as a major limitation to sustaining overwintering populations^18,24^. However, the detection of stable populations of *Ae. albopictus* in regions of Iowa with average winter temperature ranging from − 3 to − 5 °C argue that these mosquito populations have potentially adapted to survive these harsh winter conditions. Over the last decade, no discernable differences in winter temperatures were identified that would otherwise make Iowa more hospitable for overwintering survival, providing further support that *Ae. albopictus* populations have adapted to overwinter in Iowa. This also raises questions if winter temperatures alone are responsible for this climactic barrier, where the presence of leaf litter or snow cover may also help insulate overwintering *Ae. albopictus* eggs to enhance their survival^26–28^. Therefore, the microclimate of overwintering eggs may have a larger influence on the overwintering survival of *Ae. albopictus* than previously anticipated.

An additional consideration of winter temperatures is the importance of urban heat islands^29–31^, which may provide differences in microclimate across an urban and suburban landscape that results in warmer winter temperatures^30,31^, potentially improving *Ae. albopictus* overwintering survival^32^. This is supported by the importance of urbanized development on the consistent detection of *Ae. albopictus* in Des Moines and Lee counties, where the presence of more rural, agricultural environments had significant influence on the presence/absence of *Ae. albopictus.* Furthermore, our site in Polk County also resides in an urbanized environment. Therefore, these urban microenvironments may provide increased “insulation” from the harsh winter temperatures that have been traditionally believed to serve as an ecological barrier for *Ae. albopictus* expansion. As a result, this would explain the higher density of *Ae. albopictus* collected in urban environments, and the low density or absence of *Ae. albopictus* collected in more natural or agricultural areas.

At present, it is unclear as to the exact timing of when *Ae. albopictus* were introduced into the state. Prior to 2016, mosquito surveillance in Iowa predominantly focused on West Nile virus^33^, utilizing a trap network that was not ideal for the collection of invasive *Aedes* species and did not extend into areas that would most likely serve as points of introduction. In 2016, our initial surveillance efforts along Iowa’s southern border failed to detect *Ae. albopictus*^19^, yet in hindsight, these predominantly agricultural and less densely populated areas did not represent ideal habitats for *Ae. albopictus*. However, our trapping efforts in 2016 did include Lee County^19^, where our more focused efforts described in this study from 2017 to 2020 did result in the detection of established populations of *Ae. albopictus*. This discrepancy is most likely due to differences in trap locations in Lee County when compared between 2016 and 2017–2020, which for 2016 included only sites near the city of Keokuk, while in 2017–2020 included sites within both Keokuk and Ft. Madison. It is therefore of note that beginning in 2017 and in subsequent years, each of the trapping sites in the city of Ft. Madison were positive for *Ae. albopictus*. Yet, for Keokuk which is ~ 16 miles away, the first detection of *Ae. albopictus* wasn’t until 2018. This includes at least one site with continual trapping efforts in 2016^19^ and 2017 for which *Ae. albopictus* was later detected in 2018. These intra-county differences suggest that the introduction of *Ae. albopictus* in Lee County likely occurred prior to 2017 for Ft. Madison, while the introduction into Keokuk is more recent, potentially even during the years of our study (2017–2020) and suggests that their distribution is continuing to expand in the county. Similar to Ft. Madison (Lee County), we believe that the detection of *Ae. albopictus* in Burlington (Des Moines County) occurred prior to our trapping efforts in 2017 based on their abundance and distribution throughout the city. However, previous surveillance efforts in Des Moines County have been limited, preventing the determination of a definitive timeline for their introduction. Based on the prevalence of multiple mtDNA haplotypes in Des Moines County, multiple invasion events may have contributed to the established *Ae. albopictus* populations in the county. For both Des Moines and Lee counties, the proximity of the Mississippi River and interstate highways to the urban areas of both counties likely represents the most feasible route of introduction through freight and shipping along the waterway or from interstate transport from neighboring Illinois.

In contrast, the introduction of *Ae. albopictus* into Polk County, are inextricably tied to the tire transport industry. Due to the potential for annual infestations, which may be responsible for previous detections of *Ae. albopictus* in the county^7,8,17^, is difficult to definitively demonstrate that the populations of *Ae. albopictus* identified in Polk County are of established populations and not an annual infestation. Yet, during the course of our study (2017–2020), there is strong evidence that we may have captured a local infestation that was able to overwinter and establish in the area. Support for this includes an ~ tenfold increase in the number of *Ae. albopictus* collected between 2017 and 2018 (35 and 321 respectively), a dramatic increase in the percentage of *Ae. albopictus* in overall gravid *Aedes* trap yields (21% in 2017 compared to an average of 62% from 2018 to 2020), and the consistent detection of two predominant mtDNA haplotypes (*hap_1* and *hap_3*) between 2017 and 2018. Moreover, the recent detection of *Ae. albopictus* at low densities at other non-focused trapping sites in close proximity to the tire facility support their potential expansion and establishment in the area.

Our mtDNA haplotype analysis also provides insight into the origins of the *Ae. albopictus* populations in Iowa, with our three most abundant haplotypes (*hap_1, 2, and 3*) previously detected in Illinois^16^. Due to the close proximity of Illinois to Iowa, of which Des Moines and Lee counties share a border, our data imply that these *Ae. albopictus* haplotypes were most likely introduced from Illinois. However, *hap_1* has been widely detected across the United States^16,22^, southeast Asia^22,23^, and Europe^22^, making the origins of the introduction of this haplotype less clear. Additional haplotypes (*hap_4-8*) were detected at much lower frequency in our analysis, of which *hap_4, 6, 7, and 8* are unique to Iowa. However, with only a handful of studies that have examined *Ae. albopictus* mtDNA haplotypes in the United States^16,22^, further resolution of the population structure of *Ae. albopictus* is required to better understand the origins of the genetic haplotypes identified in our study. With previous studies suggesting that there is heterogeneity in *Ae. albopictus* susceptibility to arbovirus infection in natural genetic populations^34–36^, it remains to be determined what influence the genetic diversity identified in our haplotype analysis may have on *Ae. albopictus* vector competence.

Additional control measures, such as the targeted use of insecticides, may be employed to suppress these established populations of *Ae. albopictus* and to prevent their further spread to other counties in the state. However, traditional mosquito control efforts have not been very effective against *Ae. albopictus*^37^, and may only slow their future expansion. This is further complicated by the lack of mosquito abatement or vector control districts in Iowa, thus limiting the capacity for organized control efforts targeting *Ae. albopictus* in established locations and in adjacent counties where further invasion is most likely. As a result, there is little to curb the future expansion of *Ae. albopictus* in Iowa other than the abundance of agricultural land that may serve as a significant ecological barrier for the further spread of this invasive mosquito species.

The resulting epidemiological impacts of the establishment of *Ae. albopictus* in Iowa remains to be determined. While *Ae. albopictus* is a competent vector of several arboviruses such as dengue virus^10,38^, chikungunya virus^39,40^, and Zika virus^11,12^, these viruses are not endemic in the United States and would require viremic travel-associated cases for local transmission, thus making the likelihood of such events very low. A more pertinent question is whether *Ae. albopictus* and its opportunistic feeding behavior^41^ may behave as a bridge vector of mosquito-borne viruses endemic to the Upper Midwest such as West Nile virus or La Crosse virus, for which it has previously been implicated^42,43^.

Together, our results provide strong evidence for the presence and establishment of *Ae. albopictus* populations in Iowa, demonstrating the further expansion of *Ae. albopictus* into the Upper Midwest region of the United States. Importantly, with consistent winter temperatures in Iowa that are below freezing, this challenges existing beliefs that these winter temperature extremes serve as the primary boundary for *Ae. albopictus* overwintering and expansion^18,23,24,26^. With the additional recent detection of *Ae. albopictus* in Wisconsin^15^, this raises an increased need for continual surveillance to monitor the further spread and expansion of *Ae. albopictus* in the Upper Midwest and other regions of the world on the northern range of the expansion of *Ae. albopictus.* Through increased urbanization and predicted climate change, the distribution of *Ae. albopictus* is only expected to further spread^3^, highlighting the increased risk of mosquito-borne disease transmission in new regions of the world.

## Methods

### Mosquito surveillance

Targeted mosquito trapping efforts were performed by Iowa State University personnel or in collaboration with local public health departments between 2016 and 2020 from mid-May through October when mosquitoes are most active in Iowa. While initial efforts in 2016 relied on the use of BG Sentinel and CDC light traps^19^, trapping from 2017 to 2020 utilized BG Sentinel 2 (BG) traps and Gravid *Aedes* Traps (GATs) (Biogents, Regensburg, Germany). These traps have proven to be highly effective in capturing *Aedes albopictus* and other *Aedes* species that utilize artificial containers for oviposition^44–48^, with the relatively low cost and little maintenance of the GATs enabling broader coverage with each respective county. BG traps were used without a carbon dioxide source, relying on human scent lures (BG-Lure, Biogents), while the GATs were equipped with sticky cards to enable mosquito collections.

Following our efforts in 2016 which targeted 9 of the 10 counties along Iowa’s southern border^19^, we expanded our trapping efforts in 2017 to incorporate all eastern border counties along the Mississippi River and counties with more densely populated cities. These counties were chosen based on their proximity to Missouri and Illinois which have established populations of *Ae. albopictus*^7,8,13,16^ and that have the highest potential for introduction via shipping/transport into more densely populated areas. In subsequent years (2018–2020), data from the previous trapping efforts allowed for more focused surveillance, reducing the overall number of participating counties. Trapping efforts were continued in each county where *Ae. albopictus* was detected, as well as in counties that represented potential sites of introduction or spread from adjacent positive counties.

### Mosquito sample processing and identification

Mosquito samples were collected three times a week from BG Sentinel traps, while GATs were collected on a weekly basis. Samples were either transported directly or shipped to Iowa State where mosquitoes were identified to species using morphological characteristics and taxonomic keys^49^. Corresponding data were recorded according to date, trap location, and trap type. *Ae. albopictus* samples were separated into site/date-specific vials and stored in an ultra-low temperature freezer at − 80 °C for later genetic analysis.

### Geographic and land cover analysis

The latitude and longitude coordinates of all trap locations were recorded and utilized to plot trapping locations using QGIS version 3.14.1. A Landsat-based, 30-m resolution land cover layer, clipped to reflect our study area, was obtained from the Multi-Resolution Land Characteristics Consortium (MRLC) National Land Cover Database (NLCD)^50^ which served as the base map and the source of all land cover output. Land cover analysis was performed for the two counties in southeast Iowa (Lee and Des Moines counties) which displayed widespread presence of *Ae. albopictus*. Trapping site locations within these counties were examined in QGIS using a 500 m radius around each surveillance site, with the zonal histogram tool to list pixel counts of each unique land cover value within that radius (buffer layer). This distance was chosen based on the limited flight range of *Ae. albopictus,* which typically does not travel far from its site of origin with a maximum flight range of ~ 500m^51–53^. Based on pixel numbers corresponding to each land cover feature, the percent land cover was calculated for each site and used to compare between locations where *Ae. albopictus* was present or absent.

### Genetic analysis

*Ae. albopictus* samples collected from site locations between 2017 and 2018 in each of the three positive Iowa counties were examined by genetic analysis. A total of 165 samples were processed using the Marriott DNA extraction procedure^33,54,55^ to isolate genomic DNA which was used as a template for PCR genotyping. Similar to other studies that have examined *Ae. albopictus* genetic haplotypes^16,22^, a fragment of the mitochondrial cytochrome c oxidase subunit 1 was targeted with the following primers: 2027F (5′-CCC GTA TTA GCC GGA GCT AT-3′) and 2886R (5′-ATG GGG AAA GAA GGA GTT CG-3′). PCR was performed using DreamTaq Green DNA Polymerase (Thermo Fisher Scientific) under the following conditions: initial denaturation 94 °C, 3 min; denaturation 94 °C, 30 s; annealing 55 °C, 30 s; extension 72 °C, 1 min for 35 cycles; and a final extension 72 °C, 6 min. PCR products were examined by electrophoresis on a 1% agarose gel, excised, and recovered using a Zymoclean Gel DNA Recovery Kit (Zymo Research). Resulting DNA was cloned into a pJET 1.2/blunt cloning vector using the CloneJET PCR Cloning Kit (Thermo Fisher Scientific), and subsequently transformed into DH5-alpha competent *E. coli* (New England Biolabs). Bacteria were plated on LB agar plates with a 100 µg/ml ampicillin concentration and incubated overnight at 37 °C to select for successfully transformed colonies. Individual colonies were randomly chosen from the selection plates, suspended in 3 ml of LB broth and cultured overnight in a 37 °C shaker at 215 RPM. Plasmid DNA was isolated using the GeneJet Plasmid Miniprep Kit (Thermo Fisher Scientific), with the presence of an insert validated by *Bgl*II digests and gel electrophoresis. Sanger sequencing of the resulting samples was conducted by the Iowa State University DNA Facility.

DNA sequencing data was aligned and edited manually using BioEdit version 7.0.5.3. To minimize the possibility of polymerase error, at least 3 sequences from each individual sample were combined to create a consensus sequence for each sample. Any unique sequences were confirmed by the additional amplification using Phusion High-Fidelity DNA Polymerase (Thermo Fisher Scientific) followed by cloning and sequencing using the above methods. DNA from individual mosquito samples were grouped into haplotypes where each haplotype represents a unique sequence, and the number of polymorphic sites, haplotype diversity (*Hd*), and nucleotide diversity (π) were calculated using DnaSP (version 6.12.03)^16,22,56^. A haplotype network was created in PopART^57^ using the median–joining network method^58^ to visualize genetic relationships between haplotypes and to display differences in population structure between sites^16^.

## Supplementary Information

Supplementary Information 1.

Supplementary Information 2.

Supplementary Information 3.

Supplementary Information 4.

Supplementary Information 5.

Supplementary Information 6.
